# LRRC15 promotes osteogenic differentiation of mesenchymal stem cells by modulating p65 cytoplasmic/nuclear translocation

**DOI:** 10.1186/s13287-018-0809-1

**Published:** 2018-03-09

**Authors:** Yuejun Wang, Yunsong Liu, Min Zhang, Longwei Lv, Xiao Zhang, Ping Zhang, Yongsheng Zhou

**Affiliations:** 1 Department of Prosthodontics, Peking University School and Hospital of Stomatology, 22 Zhongguancun South Avenue, Haidian District, Beijing, 100081 China; 2 National Engineering Lab for Digital and Material Technology of Stomatology, Peking University School and Hospital of Stomatology, Beijing, 100081 China

**Keywords:** LRRC15, Nuclear factor κB, Osteogenic differentiation, Osteoporosis

## Abstract

**Background:**

Mesenchymal stem cells (MSCs) are a reliable resource for bone regeneration and tissue engineering, but the molecular mechanisms of differentiation remain unclear. The tumor antigen 15-leucine-rich repeat containing membrane protein (LRRC15) is a transmembrane protein demonstrated to play important roles in cancer. However, little is known about its role in osteogenesis. This study was to evaluate the functions of LRRC15 in osteogenic differentiation of MSCs.

**Methods:**

Osteogenic-induction treatment and the ovariectomized (OVX) model were performed to investigate the potential relationship between LRRC15 and MSC osteogenesis. A loss-of-function study was used to explore the functions of LRRC15 in osteogenic differentiation of MSCs in vitro and in vivo. NF-κB pathway inhibitor BAY117082, siRNA, nucleocytoplasmic separation, and ChIP assays were performed to clarify the molecular mechanism of LRRC15 in bone regulation.

**Results:**

Our results first demonstrated that LRRC15 expression was upregulated upon osteogenic induction, and the level of LRRC15 was significantly decreased in OVX mice. Both in-vitro and in-vivo experiments detected that LRRC15 was required for osteogenesis of MSCs. Mechanistically, LRRC15 inhibited transcription factor NF-κB signaling by affecting the subcellular localization of p65. Further studies indicated that LRRC15 regulated osteogenic differentiation in a p65-dependent manner.

**Conclusions:**

Taken together, our findings reveal that LRRC15 is an essential regulator for osteogenesis of MSCs through modulating p65 cytoplasmic/nuclear translocation, and give a novel hint for MSC-mediated bone regeneration.

**Electronic supplementary material:**

The online version of this article (10.1186/s13287-018-0809-1) contains supplementary material, which is available to authorized users.

## Background

With their vigorous potential for self-renewal, lack of immunogenicity, and multilineage differentiation, human mesenchymal stem cells (MSCs) are an engaging and useful therapeutic tool for tissue engineering [1, 2]. MSCs can differentiate into osteoblasts under particular conditions, and when transplanted into experimental animal models these engender bone-like mineralized tissue and are capable of rehabilitating bone defects and improving bone regeneration [3–5]. Growing evidence has revealed that the final fate of MSCs is regulated by activation or repression of lineage-specific transcription factor and epigenetic factors [6, 7]. However, the scarcity of knowledge on the molecular mechanism underlying lineage differentiation has restricted further development of MSC-based clinical applications. So, it is critical to gain a better understanding of molecular mechanisms regulating osteogenesis of MSCs.

The transcription factor nuclear factor κB (NF-κB) is defined as a pivotal regulator involved in a diversity of processes ranging from inflammation and immunity to cell survival and differentiation [8–10]. The extensive involvement of Rel/NF-κB transcription factors in bone homeostasis establishes them as targets for the regulation of osteogenic differentiation. Several researchers have indicated that estrogen deficiency inhibited the odonto/osteogenic differentiation of dental pulp stem cells by activating the NF-κB pathway [11]. Some reports also showed that a suppressed NF-κB signal could prevent osteoporotic bone loss by preserving osteoblast function [12, 13]. Furthermore, selective inhibition targeting NF-κB exhibited blocking of the receptor activator for nuclear factor κB ligand (RANKL) signal-induced osteoclastogenesis, reversed the impaired osteogenic differentiation of MSCs, and impeded inflammatory bone disorder [14–16]. What is more, activation of NF-κB has been shown to prevent the osteogenesis capability of MSCs and postnatal bone formation in vivo [12, 17].

The type I transmembrane protein 15-leucine-rich repeat containing membrane protein (LRRC15) is a member of the LRR superfamily. The LRR family is a structural module for protein–protein and protein–matrix interactions used for molecular recognition process such as cell adhesion, signal transduction, DNA repair, and RNA processing [18, 19]. Previous studies reported that the highly conserved molecule was transcriptionally upregulated in response to proinflammatory cytokines, TNF-α, IL-1β, or IFN-γ [20, 21]. LRRC15 expression was notably increased 4.6-fold in caries-diseased pulpal tissue. Remarkably, LRRC15 was relatively abundant in mineralized tissues [20]. However, the role of LRRC15 in osteogenic differentiation of MSCs and bone formation has not been delineated. Our results demonstrated that LRRC15 was significantly induced after osteogenic differentiation, while in the MSCs from bone marrow of ovariectomized mice the expression of LRRC15 was remarkably decreased. We therefore examined the effects of LRRC15 on osteogenic differentiation, and found that LRRC15 was a positive regulator for osteogenic differentiation both in vitro and in vivo. Mechanistically, we found that LRRC15 functioned as a repressor of NF-κB by promoting the nuclear exclusion of p65. We further confirmed that LRRC15 regulated osteogenic differentiation in a p65-dependent manner. Overall, these findings point to a novel important function of LRRC15 and give a novel hint for MSC-mediated bone regeneration.

## Methods

### Cell cultures and osteogenic induction

Primary human adipose-derived mesenchymal stem cells and bone marrow mesenchymal stem cells were purchased from ScienCell Research Laboratories (Carlsbad, CA, USA). All cell-based in-vitro studies were repeated in triplicate using MSCs from three donors.

Bone marrow tissues were collected from Sham and ovariectomized (OVX) mice through flushing the femurs and tibias with DMEM alpha modified Eagle’s medium (Invitrogen, Carlsbad, CA, USA). The bone marrow suspension was concentrated, washed twice in DMEM alpha modified Eagle’s medium containing 10% fetal bovine serum (FBS; Invitrogen), 2 mmol/L glutamine, 100 U/ml penicillin, and 100 μg/ml streptomycin (Invitrogen), and then cultured at 37 °C with 5% CO_2_. Cells were collected after about 2 weeks when they had expanded. MSCs were grown in a humidified incubator under 5% CO_2_ at 37 °C in DMEM alpha modified Eagle’s medium (Invitrogen) supplemented with 10% FBS (Invitrogen), 2 mmol/L glutamine, 100 U/ml penicillin, and 100 μg/ml streptomycin (Invitrogen). For osteogenic differentiation, cells were cultured in osteogenic medium (OM), which consisted of DMEM alpha modified Eagle’s medium with 10% (*v/*v) FBS, 1% (v/v) antibiotics, 100 nM dexamethasone, 10 mM β-glycerophosphate, and 0.2 mM l-ascorbic acid. Human embryonic kidney 293 T cells were maintained in complete DMEM medium with 10% FBS (Invitrogen), 100 U/ml penicillin, and 100 μg/ml streptomycin (Invitrogen). For TNF-α (R&D Systems, Minneapolis, MN, USA) or BAY117082 (Selleck, Houston, TX, USA) treatment, MSCs were starved for 24 h to synchronize the cells in the DMEM alpha modified Eagle’s medium without serum, and then changed to routine culture medium and treated with TNF-α or BAY117082.

### Plasmid and lentivirus infection

The plasmid pcDNA3-LRRC15-hemagglutinin (HA) was kindly provided by Prof. Kevin Ryan (Cancer Research UK Beatson Institute) with kind permission from Prof. Daniel Haber (Harvard Medical School). For plasmid transient infection, MSCs at 80% confluency were transfected with the pcDNA3-LRRC15-HA or pcDNA3 plasmids using the Lipofectamine 3000 transfection kit (Invitrogen) according to the manufacturer’s instructions. Viral packaging was prepared according to the manufacturer’s protocol (Clontech Laboratories, Addgene). The recombinant lentiviruses targeting *LRRC15* were purchased from GenePharma and used for MSC infection at an MOI of 100. Lentivirus infection was performed as described previously [22, 23]. For viral infections, MSCs were plated overnight, and then infected with lentiviruses in the presence of polybrene (6 μg/ml; Sigma-Aldrich, St. Louis, MO, USA) for 6 h. After 48 h, infected cells were selected with puromycin (1 mg/ml; Sigma-Aldrich, USA). The transduction efficiency was evaluated by determining the percentage of GFP-positive cells observed under an inverted fluorescence microscope (TE2000-U; Nikon).

The shRNA sequences were as follows: Scramsh, TTCTCCGAACGTGTCACGT; *LRRC15*sh1, GCACATCACTGAACTCAATGA; and *LRRC15*sh2, GCCGCAATCAGATCAGCTTCA.

### RNA interference and transient infection

Small interfering RNAs (siRNAs) targeting p65 and NC were purchased from GenePharma. For transient infection, cells were cultured and grown to 70–90% confluence, and then transfected with siRNAs using Lipofectamine 3000 (Invitrogen) according to the manufacturer’s procedure. After 48 h, cells were harvested for RNA and protein analyses. For osteogenic differentiation, cells were cultured in OM and harvested after 1 week.

The siRNA sequences were as follows: siNC, ACGUGACACGUUCGGAGAAT; and sip65, GAGUCAGAUCAGCUCCUAA.

### ALP activity and ALP staining

MSCs were grown in proliferation medium (PM) and OM. After 5 days of induction, ALP activity was assayed with an ALP activity kit using 5 μl protein lysate according to the manufacturer’s protocol (Sigma-Aldrich, USA). Signals were normalized based on protein concentrations.

After 1 week, cells were washed with PBS and then fixed in 4% paraformaldehyde at room temperature (RT) for 15 min. Subsequently, the cells were washed in PBS, incubated with a 5-bromo-4-chloro-3-indolyl phosphate–4-nitro blue tetrazolium (BCIP/NBT) staining kit (CWBIO, Beijing, China) solution for 10 min at RT, and then rinsed with water.

### Alizarin Red staining and quantification

Cells were induced for 3 weeks in osteogenic medium, fixed with 95% ethanol, and stained with 2% Alizarin Red (Sigma-Aldrich, USA). To quantitatively determine calcium, Alizarin Red was destained with 10% cetylpyridinium chloride in 10 mM sodium phosphate for 25 min at room temperature. The concentration was determined by measuring the absorbance at 562 nm on a multiplate reader and comparing to a standard calcium curve with calcium dilutions in the same solution. The final calcium level in each group was normalized to the total protein concentration detected in a duplicate plate.

### Von Kossa staining

Cells cultured in osteogenic medium for 6 weeks were assayed using the VK staining method as described previously [23, 24]. In brief, cells were fixed with 4% paraformaldehyde for 30 min at room temperature and then incubated with 5% silver nitrate solution for 1 h by exposure to a 50-W UV lamp. Unincorporated silver nitrate was removed using 5% sodium thiosulfate and rinsed three times with distilled water.

### Cell proliferation assays

MSCs were seeded at a density of 1 × 10^4^ cells/plate in 60-mm plates. Cells were counted 1, 3, 5, and 7 days after seeding with cell counting, and trypan blue dye liquor was added to the cell suspension to exclude nonviable cells. In addition, cells were also evaluated by cell counting assay using a Cell Counting Kit-8 (CCK8; Dojindo Laboratories, Kumamoto, Japan). The relative cell number was determined using CCK8 after 1, 2, 3, 4, 5, 6, and 7 days of culturing, according to the manufacturer’s instructions. The OD (absorbance) value of each well was converted to the relative cell number using a standard curve.

### Real-time reverse transcriptase-polymerase chain reaction

Total cellular RNA was isolated from MSCs using Trizol reagents (Invitrogen). From 1 μg RNA aliquots, we synthesized cDNA using random hexamers or oligo (dT), and reverse transcriptase, following the manufacturer’s protocol (Invitrogen). Real-time reverse transcriptase-polymerase chain reactions (real-time RT-PCRs) were performed using the Power SYBR Green PCR Master Mix (Roche) and a 7500 Real-Time PCR Detection System (Applied Biosystems). Glyceraldehyde-3-phosphate dehydrogenase (GAPDH) was used as an internal control. The primer sequences were as follows: *GAPDH*, (forward) 5′-CGGACCAATACGACCAAATCCG-3′ and (reverse) 5′-AGCCACATCGCTCAGACACC-3′; *OSX*, (forward) 5′-CCTCCTCAGCTCACCTTCTC-3′ and (reverse) 5′-GTTGGGAGCCCAAATAGAAA-3′; *RUNX2*, (forward) 5′-TCTTAGAACAAATTCTGCCCTTT-3′ and (reverse) 5′-TGCTTTGGTCTTGAAATCACA-3′; *ALP,* (forward) 5′-GACCTCCTCGGAAGACACTC-3′ and (reverse) 5′-TGAAGGGCTTCTTGTCTGTG-3′; *OCN*, (forward) 5′-AGCAAAGGTGCAGCCTTTGT-3′ and (reverse) 5′-GCGCCTGGGTCTCTTCACT-3′; *IL6*, (forward) 5′-CGCAACAACTCATCTCATTCTGCG-3′ and (reverse) 5′-CATGCTACATTTGCCGAAGAGC-3′; *IL8*, (forward) 5′-CGGATAAAGGGCCAAGAGAATATCCG-3′ and (reverse) 5′-TCACATTCTAGCAAACCCATTCAA-3′; *TRAF1*, (forward) 5′-CGGTGCTCTTGATCCCTACTCACCG-3′ and (reverse) 5′-GAATGGCTGCATCTCATGCTCT-3′; *CAIP2*, (forward) 5′-CAACAGATCTGGCAAAAGCA-3′ and (reverse) 5′-ATTTTCCACCACAGGCAAAG-3′; *DMP1*, (forward) 5′-CGTGGACAAAGAAGATAGCAACTCCACG-3′ and (reverse) 5′-TTCCGGCTCTCTATCTCAATGTTT-3′; *LRRC15*, (forward) 5′-GCCTTTGGACAAGGCTATGC-3′ and (reverse) 5′-GAGCAGGTACACTCGCTAGG-3′; *p65*, (forward) 5′-CCAACAACAACCCCTTCCAAGAAGA-3′ and (reverse) 5′-CGCACTGTCACCTGGAAGCA-3′; *IL1A*, (forward) 5′-TGCTGCTGAAGGAGATGCCTG-3′ and (reverse) 5′-TGCCGTGAGTTTCCCAGAAGAA-3′; *mGapdh*, (forward) 5′-AGCCCAGAACATCATCCCTG-3′ and (reverse) 5′-CACCACCTTCTTGATGTCATC-3′; and *mLrrc15*, (forward) 5′-CTGCAGTCTTGAGCCGGTCC-3′ and (reverse) 5′-GCATGGCCAGCAGCTCCA-3′. The expression of genes was calculated by the 2–^ΔΔCt^ method, and the data are shown as mean ± SD of three independent experiments.

### Western blot analysis

Cells were lysed in RIPA buffer (10 mM Tris–HCl, 1 mM EDTA, 1% sodium dodecyl sulfate (SDS), 1% NP-40, 1:100 proteinase inhibitor cocktail, 1:100 PMSF, 50 mM β-glycerophosphate, 50 mM sodium fluoride). Thirty micrograms of protein from each sample was used for western blot analysis. The samples were separated on a 10% SDS polyacrylamide gel and transferred to polyvinylidene difluoride (PVDF) membranes. The membranes were blotted with 10% dehydrated milk for 1 h, and primary antibodies against RUNX2, OSX, p65, p-p65 (Ser536), p-IκBα (ser32/ser36), tubulin, GAPDH, and PCAF (Cell Signaling Technology) and a-p65 (K310), LRRC15, OCN, and IκBα (Abcam) were diluted 1:1000 and incubated with the membranes at 4 °C overnight. Horseradish peroxidase-conjugated anti-rabbit or anti-mouse secondary antibodies (Cell Signaling Technology) were diluted 1:5000 and incubated with the membranes at room temperature for 1 h. The membranes were then visualized using an ECL kit (CWBIO). Band intensities were quantified using ImageJ software, and the signal of each target band was normalized to that of the GAPDH, tubulin, or PCAF band.

### Immunofluorescence staining

Immunofluorescence staining was performed as described previously [24]. Cells grown on sterile glass coverslips were fixed with 4% paraformaldehyde for 15 min, permeabilized with 0.25% Triton X-100 for 10 min, and then blocked with 0.8% BSA for 60 min. Then, cells were incubated with primary antibodies against osteocalcin (p65; Cell Signaling Technology) at 4 °C overnight, and incubated with the specified secondary antibodies for 1 h. Nuclei were counterstained with DAPI, and the coverslips were mounted on a glass slide. Images were captured with a LSM 5 EXCITER confocal imaging system (Carl Zeiss, Oberkochen, Germany).

### Analyses of bone formation in vivo

This study was approved by the Institutional Animal Care and Use Committee of the Peking University Health Science Center (LA2014233), and all animal experiments were performed in accordance with the Institutional Animal Guidelines. The implantation study was performed as described previously [22, 23]. The third passage of MSCs infected with lentivirus (LRRC15 or NC) was cultured in PM before in-vivo experiments. After being trypsinized and resuspended in DMEM alpha modified Eagle’s medium, 2 × 10^6^ cells were incubated with 40 mg synthograft (β-tricalcium phosphate; Bicon) for 1 h at 37 °C, followed by centrifugation at 150 × *g* for 5 min, and implanted into two symmetrical sites on the dorsal subcutaneous space of 6-week-old BALB/c homozygous nude (nu/nu) mice (*n* = 6 per group). Specimens were harvested 8 weeks after implantation, and the animals were euthanized by CO_2_ asphyxiation. The specimens were fixed in 4% paraformaldehyde and then decalcified in 10% EDTA (pH 7.4) for 2 weeks, followed by dehydration and embedding with paraffin. Sections (5-mm thickness) were cut and stained with hematoxylin and eosin (H&E) and Masson’s trichrome straining. Meanwhile, immunohistochemical staining was performed with a primary antibody against OCN (Abcam) to investigate osteogenesis. For quantification of bone-like tissue, 10 images of each sample were taken randomly (Olympus, Tokyo, Japan) and SPOT 4.0 software (Diagnostic Instruments, Sterling Heights, MI, USA) was used to measure the area of new bone formation versus total area.

### Micro-CT analyses of mice

Mice were maintained in a pathogen-free facility on a 12-h light/dark cycle with water and food provided *ad libitum*. Five-month-old mice (15 mice per group) underwent sham (Sham) or ovariectomized (OVX) operation, and the OVX mice presented osteoporosis as described previously [22, 23]. Mice underwent 3 months recovery from the ovariectomy surgery, and were then euthanized for the related assays. The proximal femur and tibia thoroughly dissected free of soft tissue was fixed with 4% paraformaldehyde for 1 day and then washed with 10% sucrose solution. Twelve hours later, images were scanned at a resolution of 8.82 mm, with tube voltage of 80 kV, tube current of 500 mA, and exposure time of 1500 ms. A typical examination consisted of a scout view, selection of the examination volume, automatic positioning, measurement, offline reconstruction, and evaluation. Two-dimensional images were used to generate 3D reconstructions using multimodal 3D visualization software (Inveon Research Workplace; Siemens, Munich, Germany) supplied by the microcomputed tomography (mCT) system.

To evaluate the mass and microarchitecture in bone between different groups, mCT was undertaken using an Inveon MM system (Siemens). Images were acquired at an effective pixel size of 8.82 mm, voltage of 80 kV, current of 500 mA, and exposure time of 1500 ms in each of the 360 rotational steps. Parameters were calculated using an Inveon Research Workplace (Siemens) as follows: bone volume/total volume (BV/TV), trabecular thickness (Tb.Th), trabecular number (Tb.N), and trabecular separation (Tb.Sp) in the trabecular region (1–2 mm distal to the proximal epiphysis) according to guidelines set by the American Society for Bone and Mineral Research [25].

### Chromatin immunoprecipitation assay

We used a chromatin immunoprecipitation (ChIP) assay kit (Merck Millipore, Darmstadt, Germany) following the manufacturer’s protocol. Briefly, 2.0 × 10^7^ cells were crosslinked with 1% formaldehyde, resuspended in lysis buffer (1% SDS, 10 mM EDTA, and 50 mM Tris–HCl (pH 8.0)) on ice for 3 min, and fragmented by sonication in RIPA ChIP buffer (0.5 mM EGTA, 140 mM NaCl, 10 mM Tris–HCl, pH 7.5, 1% Triton X-100, 0.01% SDS, 1 mM EDTA, and protease inhibitor). For DNA precipitation, soluble chromatin was then diluted and subjected to immunoprecipitation with the indicated 2 μg antibodies. Then, the immune complexes were precipitated with Protein A/G dynabeads, and washed sequentially with RIPA ChIP and TE buffer (50 mM NaCl, 5 mM EDTA, and 50 mM Tris–HCl (pH 8.0)). After the crosslinking was reversed, immune complexes containing DNA were purified and eluted. The precipitated DNA was subjected to RT-qPCR analysis. Sequences of ChIP primers were as follows: *IL6*, (forward) 5′-AAGGTTTCCAATCAGCCCCA-3′ and (reverse) 5′-TTCTCTTTCGTTCCCGGTGG-3′; *IL8*, (forward) 5′-CTTGAGGCATCTGTGAGGGA-3′ and (reverse) 5′-ATGAGCCCCTTGACCATGTG-3′; and *ICAM1*, (forward) 5′-ATTCAAGCTTAGCCTGGCCG-3′ and (reverse) 5′-ATTTCCGGACTGACAGGGTG-3′. Quantification data are expressed as the percentage of input DNA.

### Statistics

All statistical calculations were performed using SPSS10 statistical software. Comparisons between two groups were analyzed by independent two-tailed Student’s *t* tests, and comparisons between more than two groups were analyzed by one-way ANOVA followed by Tukey’s post hoc test. The data presented were derived from all three MSC or BMMSC strains. All data are expressed as the mean ± standard deviation (SD) of 3–10 experiments per group. *P* < 0.05 was considered statistically significant.

## Results

### LRRC15 was a potential target for osteoporosis treatment

**Fig. 1:**
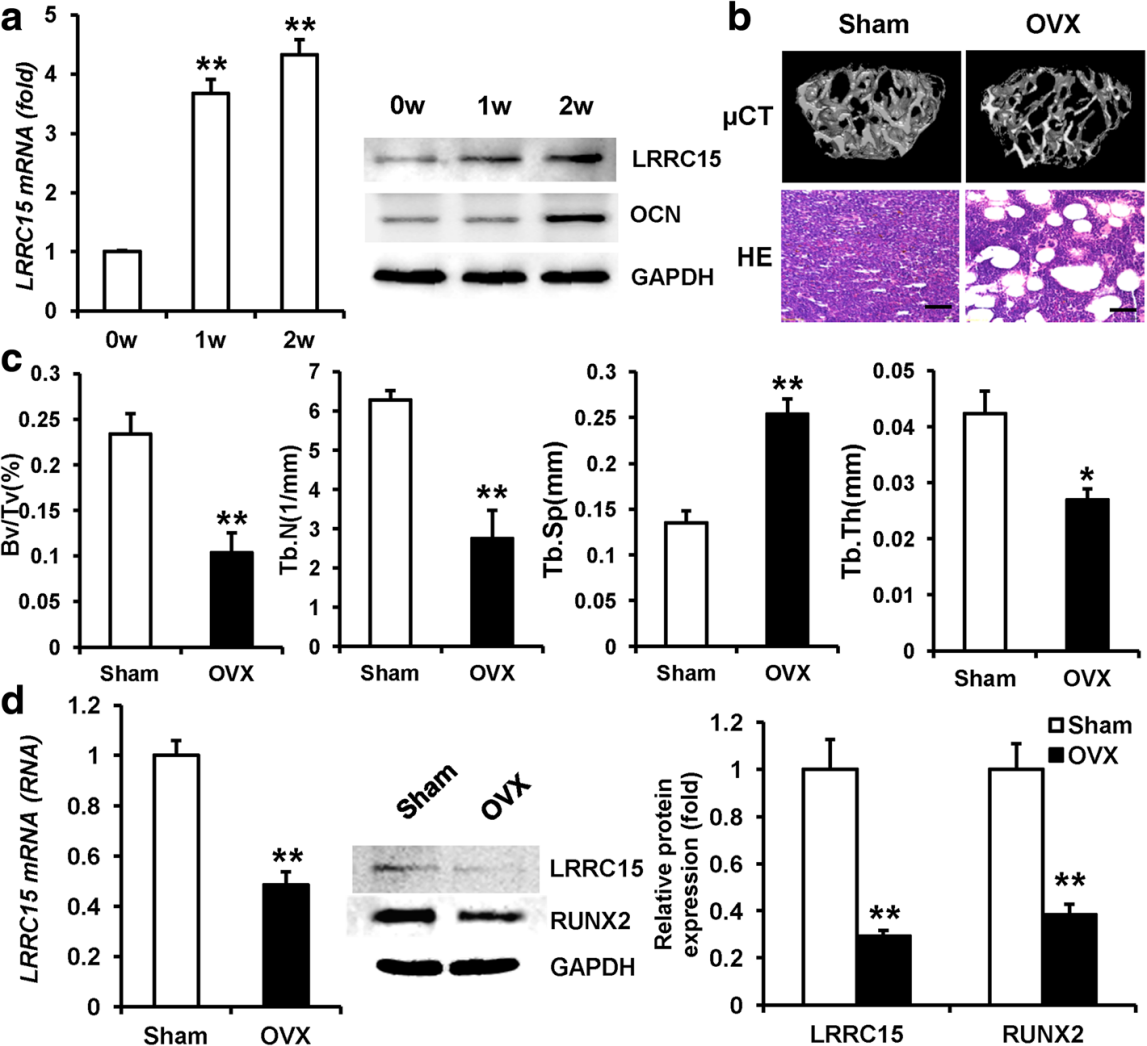
LRRC15 is a potential target for osteoporosis treatment. **a** LRRC15 expression significantly induced when MSCs undergo osteogenic induction, determined by RT-qPCR and western blot analysis respectively. **b** Representative micro-CT and H&E staining of bone loss in OVX mice. Scale bars for μCT and H&E represent 400 μm and 50 μm respectively. **c** Bone volume, trabecular number, trabecular spacing, and trabecular thickness detected in Sham and OVX mice. **d** mRNA expression of *Lrrc15* and protein levels of LRRC15 and RUNX2 tested in bone marrow-derived MSCs from OVX mice in contrast to Sham mice, determined by RT-qPCR (*left*), western blot (*middle*), and quantification (*right*) analysis. All data shown as mean ± SD, *n* = 3. **P < 0.01. w week, LRRC15 fifteen-leucine-rich repeat containing membrane protein, OCN osteocalcin, GAPDH glyceraldehyde-3-phosphate dehydrogenase, μCT microcomputed tomography, HE hematoxylin and eosin, OVX ovariectomized, Bv/Tv trabecular bone volume/tissue volume, Tb.N trabecular number, Tb.Sp trabecular spacing, Tb.Th trabecular thickness, RUNX2 runt-related transcription factor 2

First, to evaluate the potential role of LRRC15 in the process of osteogenic differentiation, we investigated expression of LRRC15 in MSCs after osteogenic induction. As shown in Fig. 1a, RT-qPCR and western blotting results showed increased expression of LRRC15 upon osteogenic induction. Next, we established the OVX mice as the animal model of postmenopausal osteoporosis. Micro-CT and HE staining showed that the trabecular bone in femurs was greatly lost in OVX compared with sham-operated mice at 3 months after operation (Fig. 1b, c). It was documented that the osteoporosis was attributed to dysfunctional osteogenic differentiation of bone marrow-derived MSCs. In order to investigate the status of LRRC15 in bone marrow MSCs of OVX mice, we examined the mRNA and protein expressions in bone marrow MSCs from Sham and OVX mice separately. As shown in Fig. 1d, the LRRC15 expression level was significantly decreased in OVX mice, with reduced expression of RUNX2 by RT-qPCR and western blot analysis.

### Knockdown of LRRC15 significantly impaired the osteogenic differentiation of MSCs in vitro

**Fig. 2:**
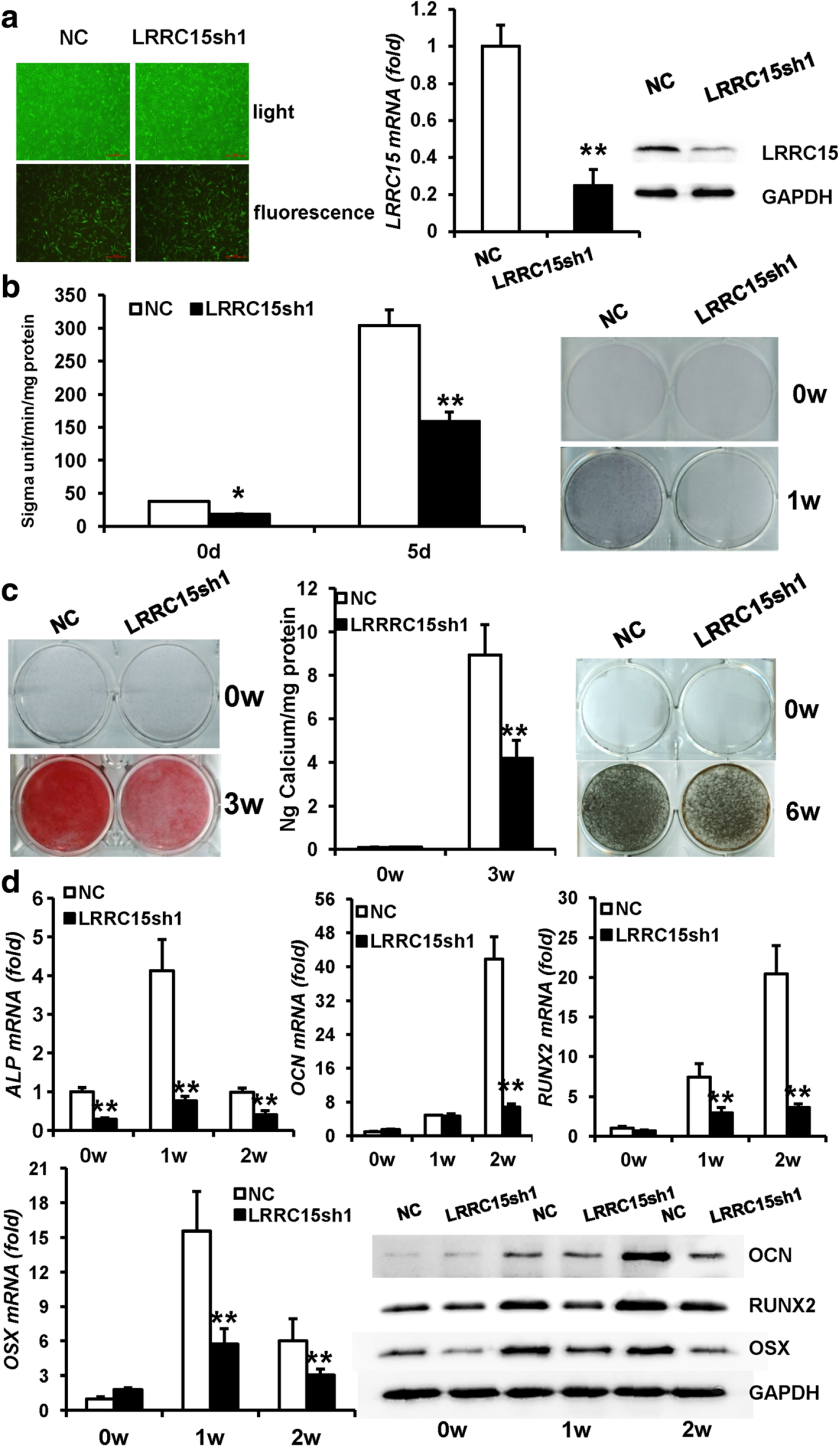
Knockdown of LRRC15 inhibits osteogenic differentiation in vitro. **a** *Left:* Microscopic images of GFP-positive MSCs under ordinary and fluorescent light. Scale bar, 500 μm. *Middle and right:* Knockdown of LRRC15 verified by RT-qPCR and western blot analysis. **b** Knockdown of *LRRC15* inhibited ALP activity (*left*) and ALP staining (*right*) in MSCs. **c** *LRRC15* knockdown restrained mineralization, shown by Alizarin Red staining (l*eft*) and calcium quantitative analysis (*middle*). *Right: LRRC15* knockdown inhibited calcium deposition, shown by Von Kossa staining. **d** mRNA expression of *ALP*, *OCN*, *RUNX2*, and *OSX* determined by RT-qPCR, and protein expression of OCN, RUNX2, and OSX determined by western blot analysis. All data shown as mean ± SD, *n* = 3. *P < 0.05; **P < 0.01. NC negative control cells, *LRRC15*sh *LRRC15* knockdown cells, LRRC15 fifteen-leucine-rich repeat containing membrane protein, GAPDH glyceraldehyde-3-phosphate dehydrogenase, d day, w week, ALP alkaline phosphatase, OCN osteocalcin, RUNX2 runt-related transcription factor 2, OSX osterix

To elucidate the function of LRRC15 in osteogenic differentiation of MSCs, we first established *LRRC15* stable knockdown MSCs using lentiviral vectors expressing a short hairpin RNA (shRNA). The GFP-positive fluorescent staining showed the majority of cells were infected by lentivirus, and the knockdown efficiency was verified by RT-qPCR and western blot analysis (Fig. 2a). Then, the cells were cultured in osteogenic medium to investigate their osteogenic differentiation potential. The results indicated that *LRRC15* knockdown strongly inhibited ALP activity and ALP staining, as shown in Fig. 2b. Moreover, the *LRRC15*sh cells showed decreased mineralization as determined by Alizarin Red staining, quantitative calcium measurements, and Von Kossa staining (Fig. 2c). RT-qPCR analysis indicated that knockdown of *LRRC15* inhibited expression of osteogenesis-related genes such as alkaline phosphatase (*ALP*), *OCN*, *RUNX2*, and osterix (*OSX*) at 0, 1, and 2 weeks after induction. Consistently, the protein levels of OCN, RUNX2, and OSX were verified by western blot analysis (Fig. 2d).

To rule out potential off-target effects, another shRNA sequence against *LRRC15* was designed. After transfection, the knockdown efficiency was verified by RT-qPCR and western blot analysis (Additional file 1: Figure S1a). When cells were cultured in osteogenic medium, we detected that, compared with negative control (NC) cells, *LRRC15* knockdown strongly inhibited ALP activity, ALP staining, Alizarin Red staining, and quantitative calcium measurements (Additional file 1: Figure S1b). Consistently, RT-qPCR results showed inhibited expressions of *ALP, OCN, RUNX2*, and *OSX* (Additional file 1: Figure S1c) in *LRRC15*sh cells compared with NC cells. Furthermore, we investigated the proliferation level of *LRRC15* knockdown cells. As shown in Additional file 2: Figure S2a, the growth curve and cell counting revealed that *LRRC15* depletion had no effects on the proliferation of MSCs. Taken together, these data suggested that LRRC15 played a critical role in osteogenic differentiation of MSCs.

### LRRC15 knockdown inhibited the osteogenic differentiation of MSCs in vivo

**Fig. 3:**
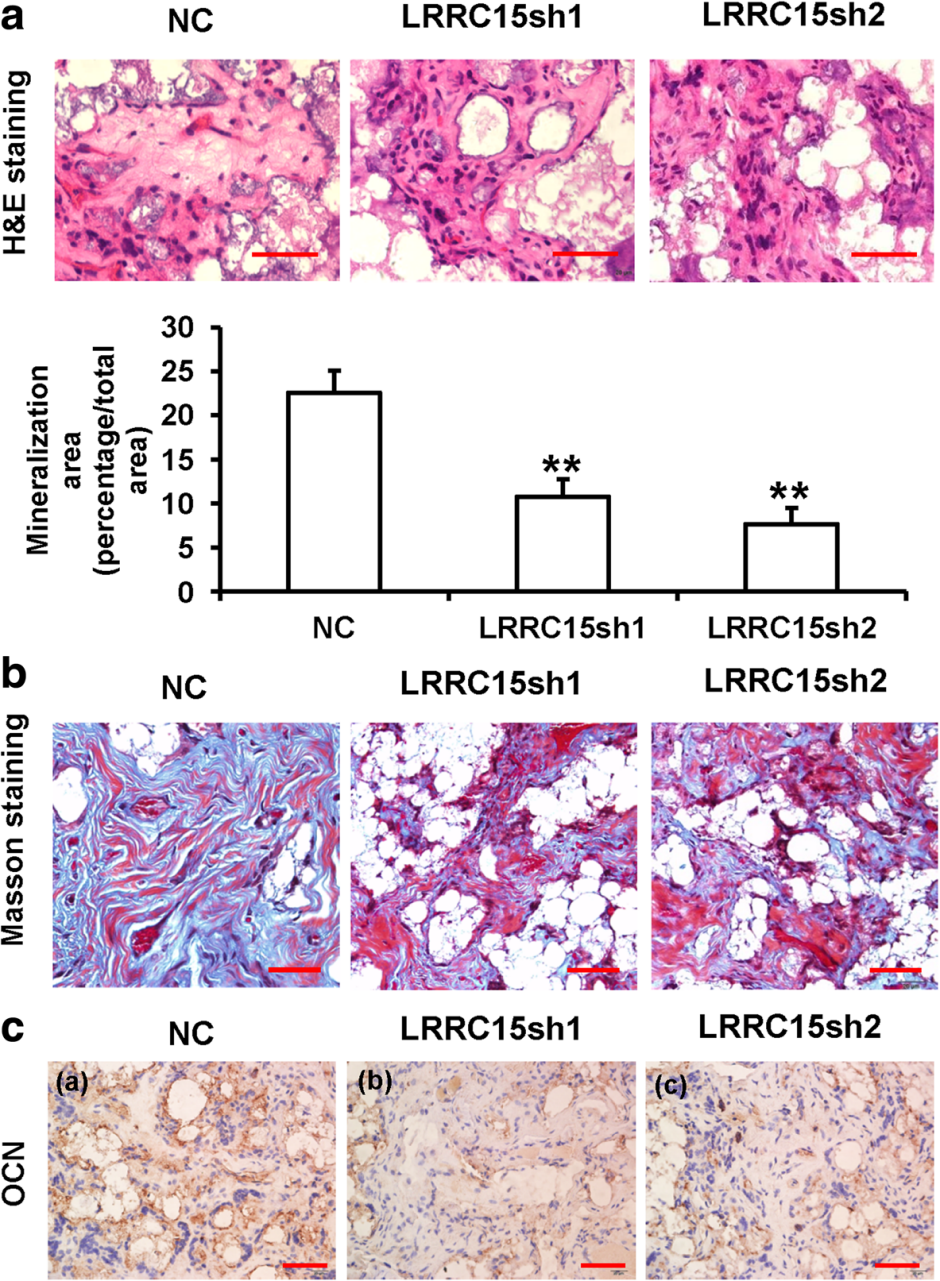
LRRC15 knockdown inhibits the osteogenic differentiation of MSCs in vivo. **A** *LRRC15* knockdown impaired MSC-mediated bone-like tissue formation in vivo, measured by H&E staining of histological sections from implanted MSC-scaffold hybrids. Scale bar represents 50 μm. Quantitative measurements of bone-like tissues showed the area of bone formation was significantly lessened in *LRRC15* knockdown cells compared with NC cells. **B** Masson’s trichrome staining of histological sections from implanted MSC-scaffold hybrids. Scale bar represents 50 μm. **C** Immunohistochemistry staining of OCN. Scale bar represents 50 μm. All data shown as mean ± SD, n = 3. **P < 0.01. NC negative control cells, H&E hematoxylin and eosin, *LRRC15*sh *LRRC15* knockdown cells, LRRC15 fifteen-leucine-rich repeat containing membrane protein, OCN osteocalcin

To further investigate the function of LRRC15 in the osteogenic differentiation of MSCs, we tested whether knockdown of *LRRC15* had an effect on MSC-mediated bone regeneration in vivo. The *LRRC15* knockdown or NC cells were mixed with matrix material and then transplanted into the dorsal sides of nude mice. After 8 weeks, the samples were harvested and underwent histological analysis. The H&E staining results demonstrated that *LRRC15*sh cells generated much less bone-like tissues compared with NC cells, and quantitative measurements inferred that knockdown of *LRRC15* significantly reduced bone-like tissue formation in vivo (Fig. 3A). Collagen deposition, as estimated by Masson staining, showed that less bone matrix was found in *LRRC15*sh/MSCs compared with the NC cells (Fig. 3B). Immunohistochemical (IHC) staining for OCN indicated that the quantity and intensity of positive staining were impaired in the *LRRC15* knockdown cells (Fig. 3C). As a whole, these results indicated the novel role of LRRC15 in bone formation in vivo.

### Knockdown of LRRC15 promoted the translocation of p65 from the cytoplasm to the nucleus

**Fig. 4:**
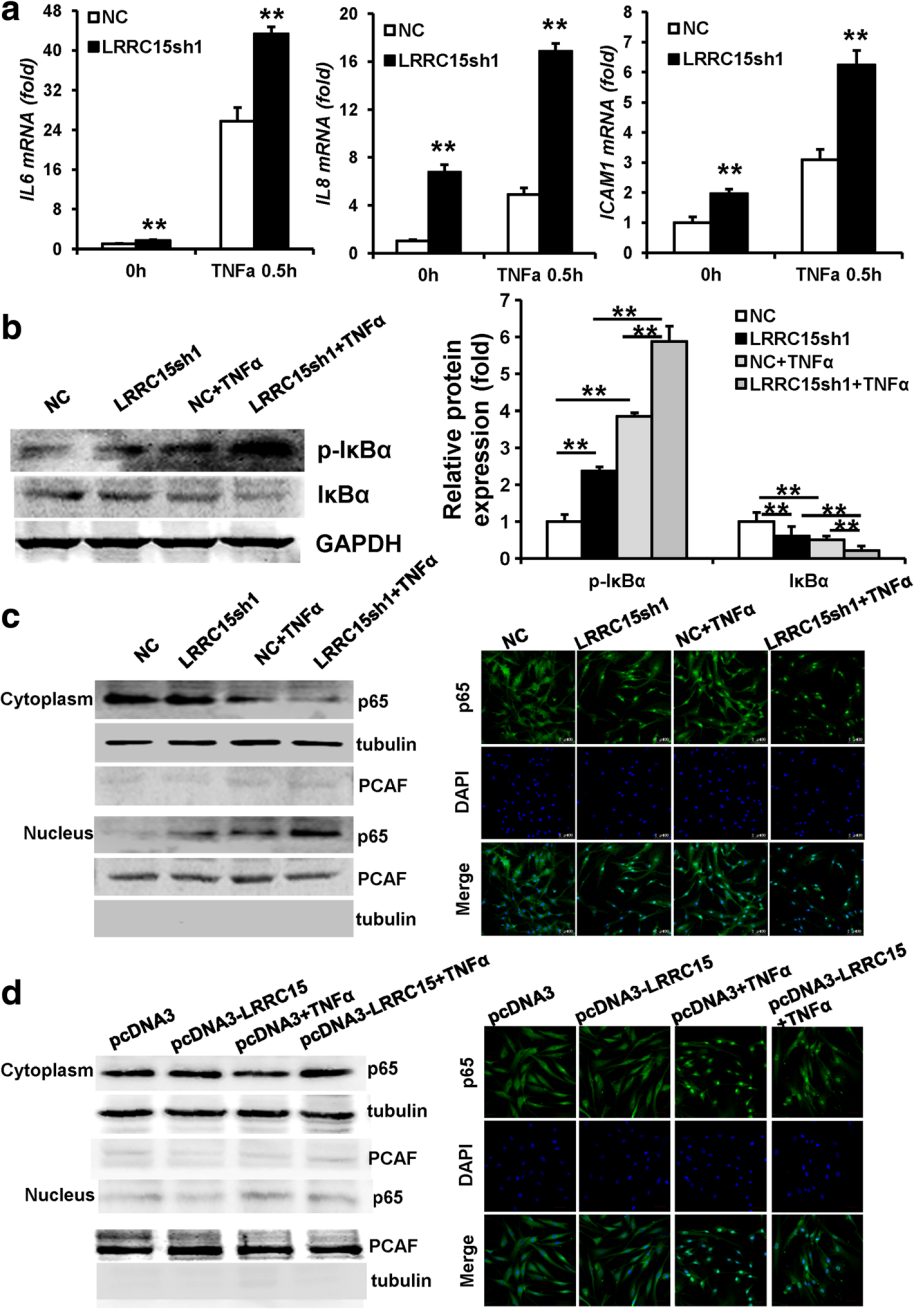
Knockdown of LRRC15 promotes translocation of p65 from cytoplasm to nucleus. **a** RT-qPCR results showing expression of p65 target genes was enhanced when *LRRC15* was silenced with a short hairpin RNA. **b** Protein levels of p-IκBα and total IκBα measured by western blot and quantitative analysis in the absence and presence of TNF-α for 30 min. **c** Nuclear and cytoplasmic proteins levels of p65, tubulin, and PCAF measured by western blot analysis after subcellular fractionation in *LRRC15*sh cells untreated or treated with TNF-α for 30 min. Cellular localization of endogenous p65 also observed by confocal microscopy in both control and LRRC15 knockdown cells with or without TNF-α treatment. Scale bars 100 μm. **d** Nuclear and cytoplasmic proteins levels of p65, tubulin, and PCAF were measured by western blot analysis after subcellular fractionation in *LRRC15*-overexpresssed cells untreated or treated with TNF-α for 30 min. Cellular localization of endogenous p65 also observed by confocal microscopy in both control and LRRC15-overexpressed cells with or without TNF-α treatment. Scale bars 50 μm. All data shown as mean ± SD, n = 3. **P < 0.01. NC negative control cells, *LRRC15*sh *LRRC15* knockdown cells, LRRC15 fifteen-leucine-rich repeat containing membrane protein, IL interleukin, TNFα tumor necrosis factor alpha, ICAM intracellular adhesion molecule, GAPDH glyceraldehyde-3-phosphate dehydrogenase, DAPI 4',6-diamidino-2-phenylindole

In order to clarify molecular mechanism by which LRRC15 promoted osteogenic differentiation of MSCs, we checked several signaling pathways and key regulators involved in MSC differentiation. Interestingly, we found that the expression of p65 targets was increased in *LRRC15* knockdown cells, as shown in Fig. 4a and Additional file 3: Figure S3a, and it seemed that LRRC15 was a negative regulator for the p65 pathway. Because IκBα is a direct NF-κB target gene, we observed that silence of LRRC15 greatly induced the phosphorylation and degradation of IκBα in the presence of TNF-α (Fig. 4b). We next tested the phosphorylation and acetylation levels of p65 with or without TNF-α stimulation in LRRC15 knockdown cells. However, the phosphorylation status and acetylation status of p65 were not influenced in LRRC15sh cells (Additional file 3: Figure S3b). To further investigate the underlying mechanism, we detected the subcellular localization of p65 in LRRC15 knockdown cells. The nuclear and cytoplasmic proteins were extracted separately and western blot analysis showed that silence of LRRC15 led to accumulation of p65 in the nucleus; moreover, inflammatory factor TNF-α stimulation caused sharply declined expression of p65 in the cytoplasm and marked enrichment of p65 in the nucleus of LRRC15sh cells. Consistently, confocal microscopy showed that p65 nuclear translocation induced by TNF-α stimulation was increased in LRRC15 knockdown cells (Fig. 4c). To further confirm the role of LRRC15 in the regulation of the nuclear exclusion of p65, we next examined cytosolic/nuclear proteins in LRRC15 overexpressed cells. As shown in Fig. 4d, LRRC15 promoted the nuclear exclusion of p65 effectively by western blot analysis and confocal microscopy. Moreover, the phosphorylation and acetylation status of p65 were not influenced in LRRC15-overexpressed cells (Additional file 3: Figure S3c). Furthermore, we performed the ChIP assay to examine p65 recruitment to the promoters of the indicated targeted genes in *LRRC15* knockdown cells. As shown in Additional file 3: Figure S3d, the deficiency of LRRC15 was found to lead to increased promoter occupancy by p65. Altogether, these results demonstrated that LRRC15 inhibited the NF-κB pathway by promoting the nuclear exclusion of p65.

### LRRC15 regulated osteogenic differentiation in a p65-dependent manner

**Fig. 5:**
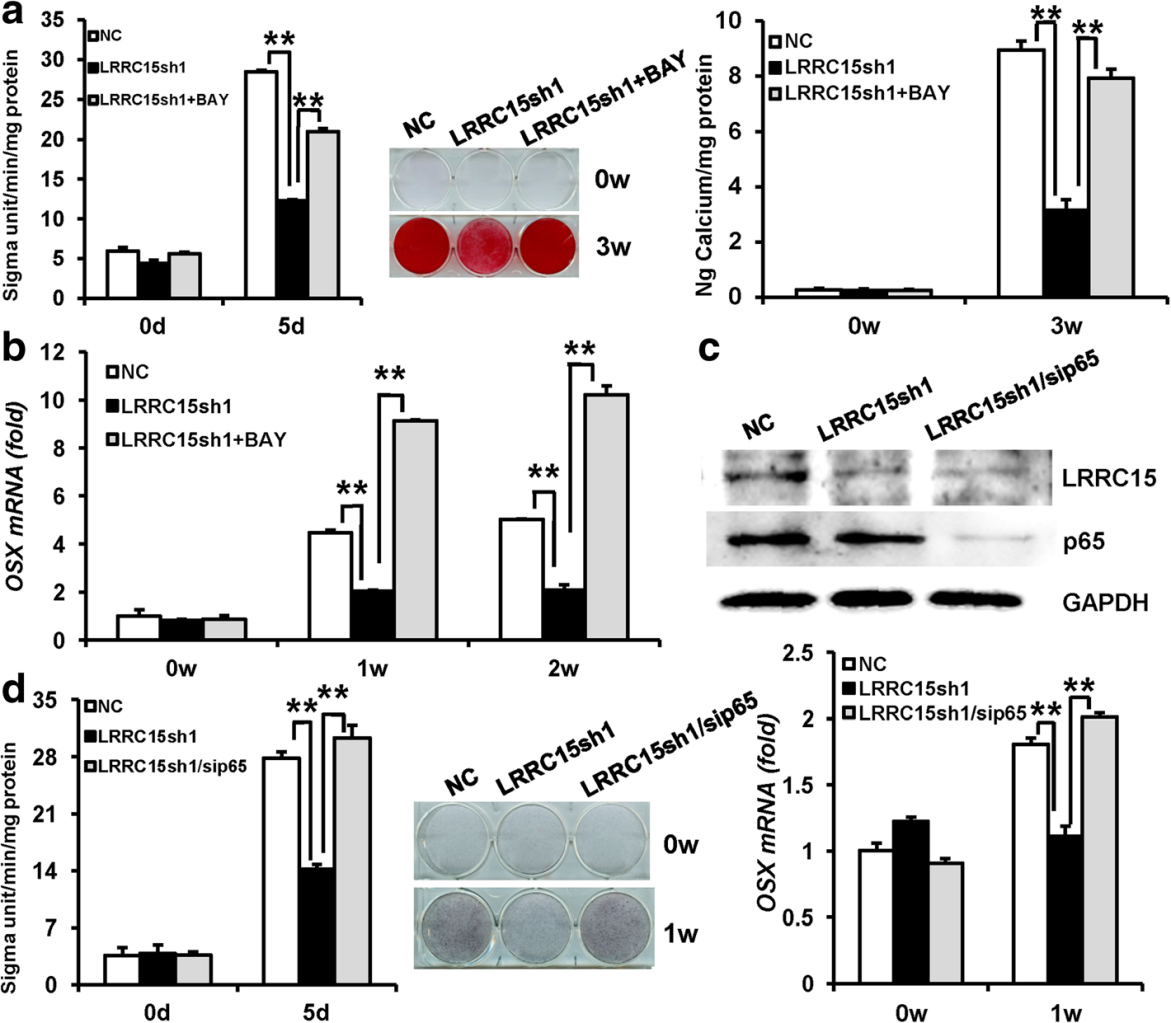
LRRC15 regulates osteogenic differentiation in a p65-dependent manner. **a** ALP activity (*left*), Alizarin Red S staining (*middle*), and quantification (*right*) in control, LRRC15 knockdown, and LRRC15 knockdown + BAY11-7082 groups. **b** RT-qPCR results showing using BAY could enhance downregulated expression of *OSX* in *LRRC15*sh cells after osteogenic induction. **c** Double knockdown of LRRC15 and p65 validated by western blot analysis. **d** ALP activity (*left*), ALP staining (*middle*), and expression of *OSX* (*right*) in control, *LRRC15* knockdown, and *LRRC15/p65* knockdown groups. All data shown as mean ± SD, n = 3. **P < 0.01. d day, w week, NC negative control cells, *LRRC15*sh *LRRC15* knockdown cells, LRRC15 fifteen-leucine-rich repeat containing membrane protein, BAY BAY117082, OSX osterix, GAPDH glyceraldehyde-3-phosphate dehydrogenase

In order to reveal the functional connection between LRRC15 and NF-κB signaling in osteogenic differentiation, we applied BAY117082 (BAY) to block the NF-κB pathway. The RT-qPCR results showed that 2 μM BAY could effectively restrain the expressions of NF-κB targeted genes, such as *IL6* and *IL8* (Additional file 4: Figure S4a). Moreover, ALP activity (Additional file 4: Figure S4b) and RT-qPCR (Additional file 4: Figure S4c) results revealed that 2 μM BAY markedly enhanced the osteogenesis in MSCs. Then, transduced MSCs were cultured in osteogenic medium with 2 μM BAY. After induction, the results showed 2 μM BAY could impactfully rescue ALP activity in *LRRC15*sh cells (Fig. 5a). Three weeks after induction, Alizarin Red staining and quantitative calcium measurements revealed marked mineralization accumulated after treatment of 2 μM BAY in *LRRC15*sh cells (Fig. 5a). Also, the RT-qPCR results showed that 2 μM BAY could promote *OSX* mRNA expression in *LRRC15* knockdown cells after induction of 1 and 2 weeks (Fig. 5b). Furthermore, we generated sip65 to block NF-κB pathway in MSCs, and the RT-qPCR and western blotting analysis (Additional file 4: Figure S4d) indicated the efficiency of p65 silencing in MSCs. The ALP activity and RT-qPCR (Additional file 4: Figure S4e) results revealed that blocking NF-κB through sip65 could also promote the osteogenic ability in MSCs. Then we used siRNA to silence *p65* in *LRRC15*sh cells. The knockdown efficiency of p65 was determined by RT-qPCR (Additional file 4: Figure S4f) and western blot analysis (Fig. 5c). The cells then underwent osteogenic induction, and the depressed osteogenic differentiation by *LRRC15* knockdown was greatly rescued in *LRRC15* and *p65* double knockdown cells, which was demonstrated by ALP activity and ALP staining (Fig. 5d). In addition, silenced *p65* could reverse the reduced expression of *OSX* (Fig. 5d) in *LRRC15* knockdown cells. As a whole, these results suggest that the novel role of LRRC15 in bone regulation was in a NF-κB-dependent manner.

## Discussion

Mouse ovariectomy has been widely used as the animal model of osteoporotic bone loss [26, 27]. Multiple studies have suggested that targeting NF-κB signaling was beneficial to treat aging-related bone loss and osteoporosis [28–30]. Previous studies found that inhibiting NF-κB signaling markedly promoted osteogenesis of MSCs in vivo and in vitro [7, 12]. Yu et al. [31] found that Wnt4 could alleviate bone loss through inhibiting NF-κB signaling in OVX and aging-related animal models. Our previous study also suggested NF-κB was a key regulator of inflammation and bone regulation, which indicated that TNF-α inhibited osteoblast differentiation by activating the NF-κB pathway [32]. Notably, our results showed that expression of LRRC15 was significantly decreased in OVX mice, which indicated LRRC15 might be the potential target for attenuating bone resorption and promoting bone formation. Moreover, silencing LRRC15 increased expression of NF-κB target genes, induced the phosphorylation and degradation of IκBα, and activated nuclear translocation of p65. In the present study, we demonstrated that LRRC15 played a prominent role in osteogenic commitment of MSCs. Both in-vitro and in-vivo experiments suggested that LRRC15 might function as a target for the treatment of metabolic bone disease such as osteoporosis through governing MSC fate. Interestingly, we demonstrated an undiscovered interaction between LRRC15 and NF-κB: LRRC15 was a novel regulator for the transcription activity of NF-κB.

The trans-activity and nuclear localization of p65 is regulated by a sophisticated network. Various environmental stimuli lead to different modifications of p65, including phosphorylation, acetylation, methylation, and ubiquitination. In a resting status, the prototypical NF-κB complex, p50/p65 heterodimer, is sequestered in the cytoplasm associating with IκBα, which exhibits the nucleocytoplasmic shuttling ability. Increasing evidence shows that, among these modifications, p65 phosphorylation owns a significant role in nuclear regulation for optimal NF-κB transcriptional activity [6, 33]. Similarly, the reversible deacetylation of p65 is also critical for governing IκBα’s binding to p65 and the nuclear export of the NF-κB complex [34]; while, different from phosphorylation, acetylation of p65 seems to be restricted to the nucleus, consistent with the nuclear localization of regulator for acetylation--p300/CBP [35]. Phosphorylation of p65 at serine 276 can raise the recruitment of coactivator p300/CBP, achieving full transcriptional activation involving p65 acetylation, and the acetylation is largely contingent on prior phosphorylation [36]. In the present study, although we did not detect obvious changes of p65 phosphorylation in *LRRC15* knockdown cells, we confirmed that *LRRC15* silencing led to the nuclear localization of p65. Besides, we observed that the acetylation level of p65 had no marked regulation in *LRRC15* knockdown cells. Besides phosphorylation and acetylation, methylation of p65 is an innovative mechanism to govern p65-DNA binding under inflammatory chemokine stimuli. Similar to acetylation, methylated p65 is confined to the nucleus and this posttranslational modification coordinates with histone modifications [37]. When these regulators enter the nucleus and bind to the promoter of p65, the remodeling machinery of local chromatin is activated. Some research indicates that PRMT5-mediated arginine methylation is required for p65 activation that facilitates the expression of stimulus-specific factor [38]. In addition, p65 can be monomethylated by histone methyltransferase Set9 at lysine 37 after inflammatory cytokine treatment [39]. In the present study, we confirmed that the nuclear accumulation of p65 in *LRRC15*sh cells was irrelevant to the phosphorylation and acetylation status of p65. Further research is extremely needed to explore whether the p65 methylation modification involved the nucleocytoplasmic shuttling mechanism.

In addition to the newly discovered interaction between LRRC15 and NF-κB, our present work, for the first time, confirmed the significant role of LRRC15 during osteogenesis. Solid data indicated that knockdown of *LRRC15* impaired the osteogenic differentiation of MSCs both in vitro and in vivo. Most importantly, we observed decreased LRRC15 expression in bone marrow MSCs from OVX mice. As we know, postmenopausal osteoporosis is caused by dysfunctional osteogenic differentiation of bone marrow-derived MSCs; we believe that our findings not only explore a new factor involved in the mediation of osteogenic differentiation of MSCs, but also have important implications for the treatment of postmenopausal osteoporosis.

## Conclusions

In summary, our results first demonstrate that LRRC15 enhanced the osteogenic differentiation potential of MSCs through repressing the NF-κB pathway by promoting the nuclear exclusion of p65. Furthermore, we found that LRRC15 regulated osteogenic differentiation in a p65-dependent manner. Collectively, this work points to a new important function of LRRC15, elucidates the molecular mechanisms underlying directed differentiation in MSCs, and gives a novel hint for MSC-mediated bone regeneration.

## Additional files

Additional file 1: **Figure S1.** Kockdown of *LRRC15* represses osteogenic differentiation in vitro. a Microscopic images of GFP-positive MSCs (*left*) under ordinary and fluorescent light. Scale bar, 500 μm. Knockdown of LRRC15 verified by RT-qPCR (*middle*) and western blot (*right*) analysis. b *LRRC15* knockdown reduced ALP activity and ALP staining (*left*). *LRRC15* knockdown inhibited mineralization, shown by Alizarin Red staining and calcium quantitative analysis (*right*). c Silence of *LRRC15* inhibited expression of *ALP*, *OCN*, *RUNX2*, and *OSX*. All data shown as mean ± SD, *n* = 3. **P < 0.01. NC negative control cells, *LRRC15*sh *LRRC15* knockdown cells, d day, w week. (TIFF 992 kb)

Additional file 2: **Figure S2.** LRRC15 knockdown has no effect on cell proliferation. a Proliferation of *LRRC15* knockdown cells determined by CCK8 (*left*) and cell counting (*right*) assays. NC negative control cells, *LRRC15*sh *LRRC15* knockdown cells. (TIFF 903 kb)

Additional file 3: **Figure S3.** a Expression of *TRAF1* (*upper*) and *cIAP2* (*lower*) in *LRRC15* knockdown cells. b Phosphorylation and acetylation of p65 in LRRC15 knockdown cells, shown by western blot and quantitative analysis. c Phosphorylation and acetylation of p65 in LRRC15 overexpressed cells measured by western blot and quantitative analysis. d ChIP analysis indicated that *LRRC15* deficiency led to increased p65 occupancy on *IL6* (*left*), *IL8* (*middle*), and *ICAM1* (*right*) promoters. All data shown as mean ± SD, *n* = 3. **P < 0.01. NC negative control cells, *LRRC15*sh *LRRC15* knockdown cells. (TIFF 330 kb)

Additional file 4: **Figure S4.** a Expression of *IL6* and *IL8* with various concentrations of BAY treatment. b ALP activity enhanced in presence of 2 μM BAY. c RT-qPCR analysis exhibited that *ALP* and *OSX* expression was significantly upregulated upon BAY treatment (2 μM). d p65 knockdown efficiency detected by RT-qPCR and western blot analysis in control or sip65-treated cells. e ALP activity and expression of *ALP* and *OSX* enhanced in sip65-treated cells after osteogenic differentiation. f Levels of *LRRC15* and *p65* in LRRC15 and p65 double knockdown cells, shown by RT-qPCR. All data shown as mean ± SD, n = 3. **P < 0.01. NC negative control cells, *LRRC15*sh *LRRC15* knockdown cells, d day, w week. (TIFF 784 kb)

## Abbreviations

ALP: Alkaline phosphatase
ARS: Alizarin Red staining
BV/TV: Bone volume/total volume
EDTA: Ethylenediamine tetraacetic acid
GAPDH: Glyceraldehyde-3-phosphate dehydrogenase
H&E: Hematoxylin and eosin
IFN-γ: Interferon gamma
IHC: Immunohistochemistry
IL: Interleukin
LRRC15: Fifteen-leucine-rich repeat containing membrane protein
mCT: Microcomputed tomography
MSC: Mesenchymal stem cell
NF-κB: Transcription factor nuclear factor κB
OCN: Osteocalcin
OM: Osteogenic medium
OSX: Osterix
PM: Proliferation medium
RANKL: Receptor activator for nuclear factor κB ligand
RUNX2: Runt-related transcription factor 2
siRNA: Small interfering RNA
Tb.N: Trabecular number
Tb.Sp: Trabecular separation
Tb.Th: Trabecular thickness
TNF-α: Tumor necrosis factor alpha
