# Supplementary figures and images for: LRRC15 promotes osteogenic differentiation of mesenchymal stem cells by modulating p65 cytoplasmic/nuclear translocation
Source: Stem Cell Res Ther. 2018 Mar 9;9:65. doi: 10.1186/s13287-018-0809-1 (PMC5845373; doi:10.1186/s13287-018-0809-1)

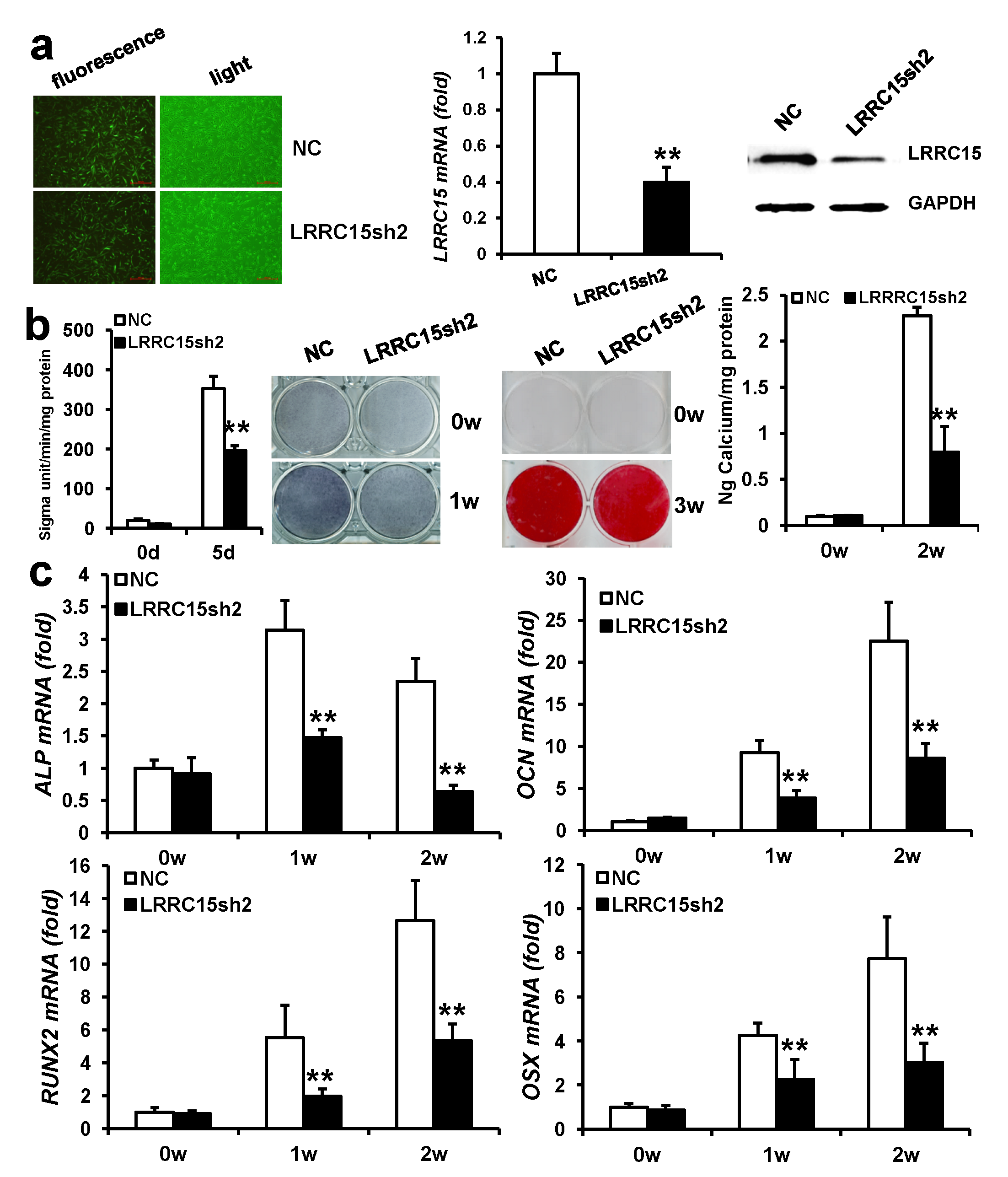

Supplement: Supplementary file 1 — Figure S1. Kockdown of LRRC15 represses osteogenic differentiation in vitro. a Microscopic images of GFP-positive MSCs (left) under ordinary and fluorescent light. Scale bar, 500 μm. Knockdown of LRRC15 verified by RT-qPCR (middle) and western blot (right) analysis. b LRRC15 knockdown reduced ALP activity and ALP staining (left). LRRC15 knockdown inhibited mineralization, shown by Alizarin Red staining and calcium quantitative analysis (right). c Silence of LRRC15 inhibited expression of ALP, OCN, RUNX2, and OSX. All data shown as mean ± SD, n = 3. **P < 0.01. NC negative control cells, LRRC15sh LRRC15 knockdown cells, d day, w week. (TIFF 992 kb) [file 13287_2018_809_MOESM1_ESM.tif]

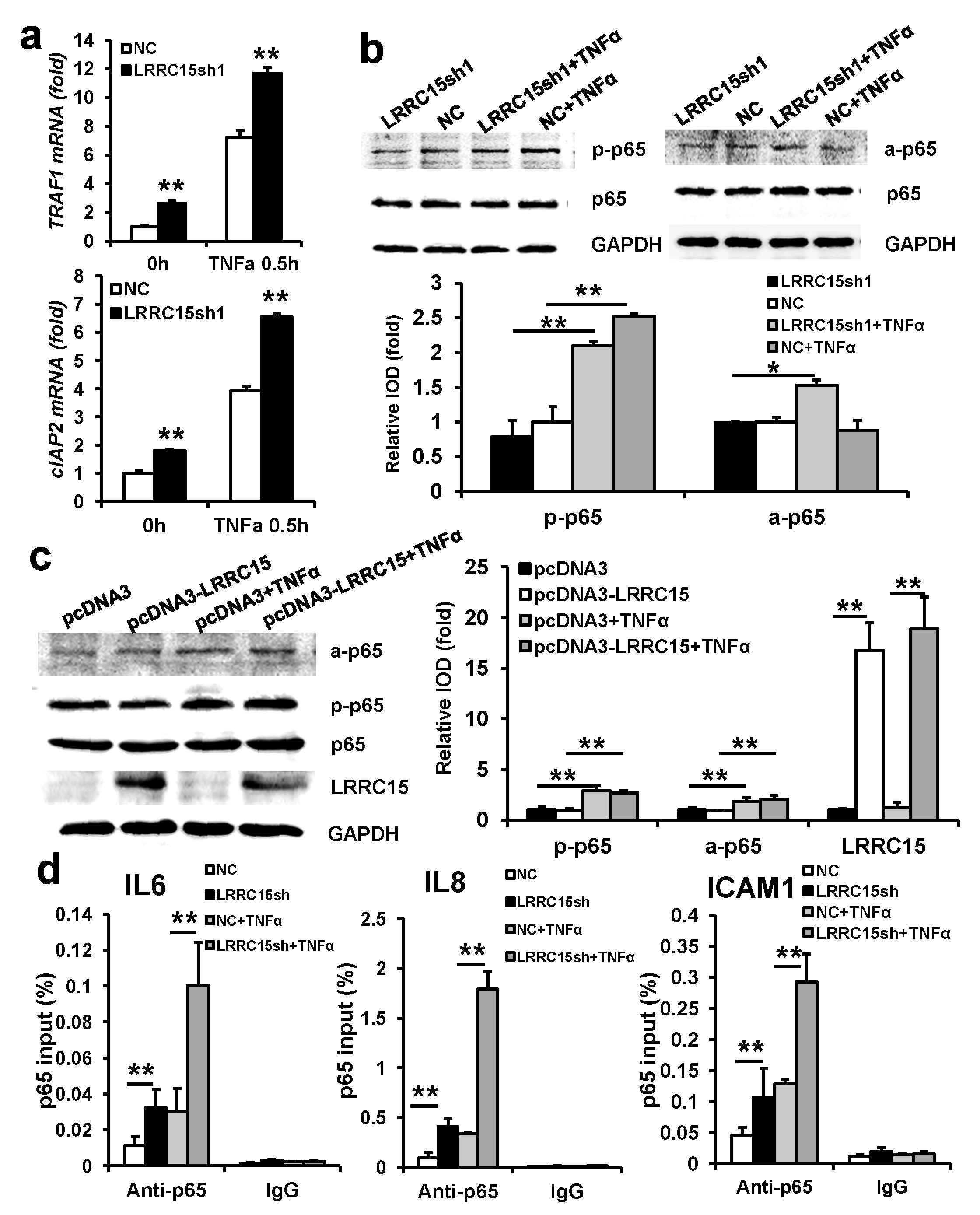

Supplement: Supplementary file 3 — Figure S3. a Expression of TRAF1 (upper) and cIAP2 (lower) in LRRC15 knockdown cells. b Phosphorylation and acetylation of p65 in LRRC15 knockdown cells, shown by western blot and quantitative analysis. c Phosphorylation and acetylation of p65 in LRRC15 overexpressed cells measured by western blot and quantitative analysis. d ChIP analysis indicated that LRRC15 deficiency led to increased p65 occupancy on IL6 (left), IL8 (middle), and ICAM1 (right) promoters. All data shown as mean ± SD, n = 3. **P < 0.01. NC negative control cells, LRRC15sh LRRC15 knockdown cells. (TIFF 330 kb) [file 13287_2018_809_MOESM3_ESM.tif]
